# A pharmacodynamic comparison of 5 anti-platelet protocols in patients with ST-elevation myocardial infarction undergoing primary PCI

**DOI:** 10.1186/1471-2261-14-189

**Published:** 2014-12-16

**Authors:** Sasha Koul, Pontus Andell, Andreas Martinsson, J Gustav Smith, Fredrik Scherstén, Jan Harnek, Matthias Götberg, Eva Norström, Sven Björnsson, David Erlinge

**Affiliations:** Department of Cardiology, Lund University, Skåne University Hospital Lund, SE 221 85 Lund, Sweden; Department of Clinical Chemistry, Skåne University Hospital Malmö, Malmö, Sweden

**Keywords:** Prasugrel, Ticagrelor, Clopidogrel, Upstream, STEMI

## Abstract

**Background:**

Despite advances in anti-platelet treatments, there still exists an early increase in both ischemic as well as bleeding events following primary PCI in patients with ST-elevation myocardial infarction (STEMI). Platelet inhibition data of different anti-platelet treatments in the acute phase of a myocardial infarction might offer some insight into these problems. The aim of this study was to evaluate the pharmacodynamic profile of 5 different anti-platelet treatments in the acute phase of STEMI in patients undergoing primary PCI.

**Methods:**

A total of 223 STEMI patients undergoing primary PCI were prospectively included. Patients received either pre-hospital clopidogrel only, pre-hospital clopidogrel followed by prasugrel switch in the cath lab, prasugrel treatment only, pre-hospital clopidogrel followed by ticagrelor switch in the cath lab or pre-hospital ticagrelor only. Platelet reactivity was measured serially using vasodilator-stimulated phosphoprotein (VASP).

**Results:**

Patients receiving pre-hospital clopidogrel followed by prasugrel switch showed similar platelet inhibition data as patients receiving prasugrel only, with more than 90% being good responders the day after PCI. Average time from prasugrel administration to a VASP value of <50% was 1.5 hours. In patients receiving pre-hospital ticagrelor, 50% were good responders at completion of PCI and average time to a VASP-value of <50% was 2.3 hours. Only 32% of patients receiving clopidogrel only were responders the day after PCI.

**Conclusions:**

Switching from an upstream bolus dose of clopidogrel to prasugrel at the time of PCI, appeared as a safe and feasible option with no tendency for overshoot or attenuation of platelet inhibition. Pre-hospital administration of ticagrelor was associated with a 50% good responder rate at completion of PCI.

## Background

Usage of P2Y12-inhibitors constitutes a cornerstone in the treatment of acute coronary syndromes, including patients with ST-elevation myocardial infarction (STEMI) [1–3]. Despite modern P2Y12-inhibitors like prasugrel and ticagrelor, there still exists an early increase in ischemic events following primary PCI in patients with STEMI. Furthermore there is also an immediate increased risk of bleeding [4]. Although guidelines recommend as early administration as possible of P2Y12-inhibitors, clinical data regarding the timing of P2Y12-inhibitor administration in STEMI patients is limited [5, 6]. Neither prasugrel nor ticagrelor have any outcome data regarding effects of pre-treatment in STEMI patients. Clopidogrel pre-treatment has in register studies and in the small randomized CIPAMI trial shown promise compared to no pre-treatment at all, however large randomized data do not exist [7–11]. Platelet inhibition data might give some insight into these questions, however pharmacodynamic data regarding prasugrel, ticagrelor and even clopidogrel in the acute phase of STEMI is also limited [12, 13]. Pre-treatment protocols with other substances, including GPIIb/IIIa-inhibitors have been studied with mixed results on various efficacy endpoints [14–16].

The aim of this study was to evaluate the platelet inhibition of 5 different anti-platelet protocols in the acute phase of STEMI in patients undergoing primary PCI.

## Methods

### Study design

**Figure 1:**
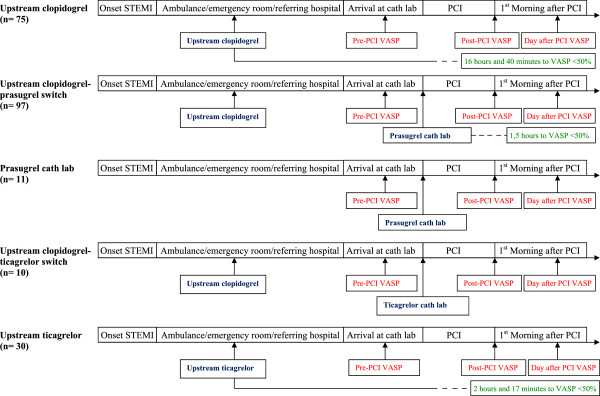
**Clinical protocol.** Flow-chart describing the 5 patient cohorts in the study and timings of blood sampling.

Patients undergoing PCI for STEMI at Skåne University Hospital in Lund were prospectively included in the Lund Platelet Registry from October 2009 to October 2012 (total n = 223). All STEMI patients were eligible for inclusion. However if the patients had not received a P2Y12-inhibitor or primary PCI was not performed, they were excluded. Aspirin treatment was given as standard treatment unless contraindicated in the individual patient. Bivalirudin was used as first-line anti-thrombotic adjuvant therapy during PCI. Usage of GPIIb/IIIa-inhibitors were used as bail-out option at the physician’s discretion (Table 1). Platelet reactivity was measured serially using a flow cytometric assay for the vasodilator-stimulated phosphoprotein (VASP) at three time-points: a) After performed angiography prior to PCI (pre-PCI VASP) b) after completed PCI procedure (post-PCI VASP) and c) the following morning after PCI (day after VASP). A total of 5 different cohorts were included according to their P2Y12-inhibition (Figure 1). 1) At the time of initiation of the registry all patients were treated with upstream clopidogrel only (*upstream clopidogrel* group, n = 75).

**Table 1:** **Patient characteristics**

	Upstream clopidogrel	Upstream clopidogrel-prasugrel switch	Prasugrel cath lab	Upstream clopidogrel-ticagrelor switch	Upstream ticagrelor	P-value
	(n = 75)	(n = 97)	(n = 11)	(n = 10)	(n = 30)	
Age	71 yrs	62 yrs	61 yrs	64 yrs	65 yrs	<0.01
Male sex	53 (71%)	75 (77%)	8 (73%)	8 (80%)	20 (67%)	0.74
Smoking status						<0.01
Never smoked	29 (39%)	23 (25%)	4 (36%)	2 (20%)	5 (17%)	
Previous smoker	28 (37%)	25 (28%)	0 (0%)	4 (40%)	6 (20%)	
Current smoker	17 (23%)	43 (47%)	6 (55%)	4 (40%)	16 (53%)	
*Adjuvant anti- thrombotic treatment*						
Aspirin	74 (99%)	91 (94%)	11 (100%)	10 (100%)	30 (100%)	0.09
Heparin	72 (96%)	90 (93%)	11 (100%)	10 (100%)	30 (100%)	0.39
GpIIb/IIIa-inhibitors	5 (6.7%)	1 (1.1%)	1 (9.1%)	0 (0%)	0 (0%)	0.13
Bivalirudin	70 (93%)	86 (89%)	11 (100%)	10 (100%)	30 (100%)	0.17
*Prior diseases*						
Hypertension	46 (61%)	28 (31%)	1 (9.1%)	2 (20%)	15 (50%)	<0.01
Myocardial infarction	15 (20%)	7 (7.7%)	1 (9.1%)	1 (10%)	3 (10%)	0.15
Diabetes	6 (8.0%)	11 (12%)	0 (0%)	2 (20%)	5 (17%)	0.42
Previous CABG	3 (4.0%)	0 (0%)	0 (0%)	0 (0%)	2 (7%)	0.17
Previous PCI	9 (12%)	7 (7.2%)	0 (0%)	1 (10%)	4 (13%)	0.59
*Insertion of drug eluting stent*	6 (8%)	10 (10%)	3 (27%)	0 (0%)	7 (23%)	0.06

GpIIb/IIIa; Glycoprotein IIb/IIIa, CABG; Coronary Arterty Bypass Grafting, PCI; Percutaneous Coronary Intervention.

2) As the results of the TRITON trial were published prasugrel was incorporated into clinical practice as a bolus dose of prasugrel (60 mg) in the catheterization laboratory on top of a previous bolus dose of upstream clopidogrel (600 mg) in patients with no major risk factors for bleeding or other contraindications (*upstream clopidogrel-prasugrel switch,* n = 97). A weight below 60 kg or age above 75 years were not considered as contraindications for a bolus dose prasugrel, however maintenance therapy for these patients consisted of clopidogrel 75 mg o.d. A history of stroke or TIA was considered an absolute contraindication for any use of prasugrel. Prasugrel was not used in a pre-hospital setting as it was not endorsed in either national or international guidelines as a pre-hospital drug at that time. 3) A subset of patients were only given prasugrel at the cath lab after performed coronary angiography (*prasugrel cath lab* group, n = 11). 4) As ticagrelor became available, patients were initially given a bolus dose of clopidogrel upstream (600 mg) followed by a bolus dose of ticagrelor (180 mg) in the catheterization laboratory (*upstream clopidogrel-ticagrelor switch,* n = 10) unless contraindicated. 5) As pre-hospital ticagrelor became available in early 2012, patients were subsequently given ticagrelor mono-therapy (180 mg) as an upstream bolus dose (*upstream ticagrelor* group, n = 30) unless contraindicated.

If patients were deemed not suitable for either prasugrel or ticagrelor they were per protocol given clopidogrel. A recommended treatment duration of at least one year of P2Y12-inhibition was recommended. Nearly all patients were given concomitant aspirin (Table 1).

### Patient data and clinical follow-up

Patient data was primarily obtained from the Swedish Coronary Angiography and Angioplasty Registry (SCAAR) as well as through hospital patient records. The SCAAR registry includes data from all centres that perform coronary angiography or PCI in Sweden. Based on the unique Swedish 10-digit personal identification number, the SCAAR register was merged with other national registries, including the Swedish Hospital Discharge Registry and The Register of Information and Knowledge about Swedish Heart Intensive care Admissions (RIKS-HIA).

### VASP-analysis

Platelet reactivity was measured using a commercially available flow cytometric assay of intraplatelet vasodilator-stimulated phosphoprotein (VASP), with analyses performed according to the manufacturer’s instructions (Biocytex Platelet VASP kit, Marseille, FR) [17]. The platelet reactivity index (VASP-PRI) was calculated from the corrected mean fluorescence intensity (cMFI) following incubation of the platelets with either prostaglandin E1 alone or prostaglandin E1 with ADP using the formula:


### Endpoints

1. Percentage of patients reaching a VASP PRI-value of <50% the day after PCI. The cut-off value for VASP-PRI was selected as a value above 50% has been associated with worse clinical outcomes following PCI [18].
2. Average time to reach a VASP PRI-value of <50%.

As safety parameter, major in-hospital bleeding events were recorded (fatal bleeding/cerebral bleeding/bleeding requiring surgery or transfusion.

### Ethics

The study protocol was in accordance with the Declaration of Helsinki. Patient informed consent and ethical approval was not needed as all blood sampling was part of routine coronary care and no blood samples were stored for further usage.

### Statistical methods

Baseline characteristics were compared across the various patient groups using ANOVA for continuous parametric data and Pearson’s chi-squared test for categorical data. Platelet reactivity as measured by continuous VASP PRI-values was compared across time-points within treatment groups using ANOVA for parametric data and Mann-Whitney’s *U*-test for non-parametric data. The equality of variances assumption was tested using Levene’s test. The proportion of patients reaching a VASP PRI < 50% was compared using Pearson’s chi-squared test. A p-value <0.05 was considered significant. The risk of major in-hospital bleeding was evaluated using the Kaplan-Meier estimator, with censoring at death or loss to follow-up. VASP sampling the day after PCI was performed on fixed times (between 05.00-06.00) but the time of the day during which PCI was performed varied (according to when then the patient arrived to the cath lab). Furthermore durations of PCI procedures varied, both factors allowing creation of time separation curves. These were created using linear regression models with VASP-PRI regressed on linear or log-transformed time, as appropriate from cluster plots. All analyses were performed using SPSS (SPSS version 18, SPSS Inc, Chicago).

## Results

Patient characteristics are summarized in Table 1. *Upstream clopidogrel* patients were on an average older and had more comorbidities like hypertension and previous myocardial infarction than the other groups, in accordance with the clinical protocol where patients not deemed eligible for ticagrelor or prasugrel would receive clopidogrel. Anticoagulation with heparin and bivalirudin was often used during PCI procedures in the study. Nearly all patients received aspirin.

### *Clopidogrel upstream*group

**Figure 2:**
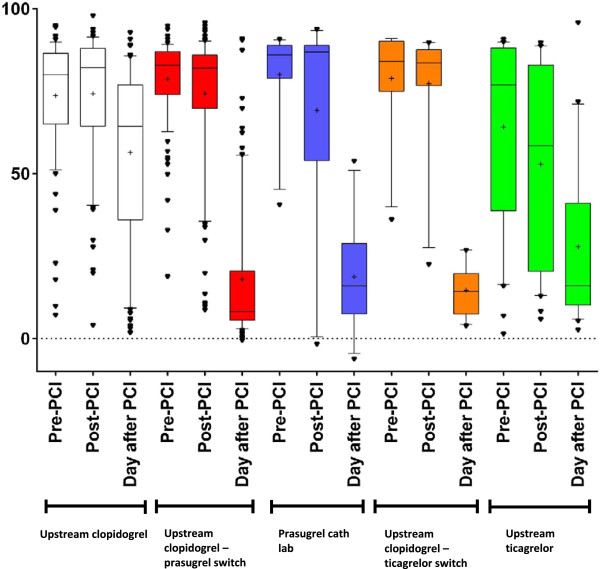
**Average VASP-PRI values.** Box-plot of median and average VASP-PRI values in the various patient cohorts (boxes denote median and 25-75 percentile and whiskers denote 10-90 percentile with outliers. Plus sign in boxes denotes the average value).

**Figure 3:**
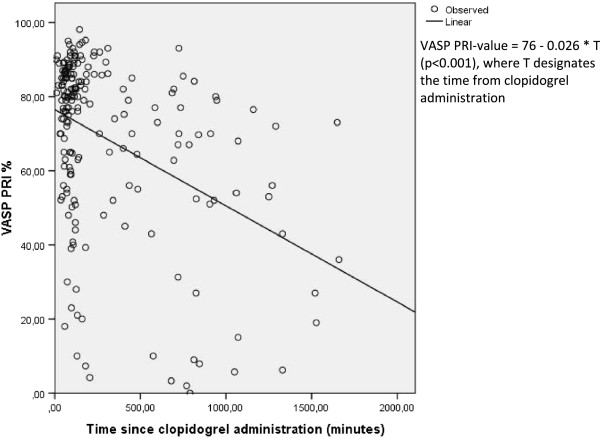
**Regression curve upstream clopidogrel.** VASP values as function of time in upstream clopidogrel patients with a linear regression plot.

Patients in the *clopidogrel upstream* cohort had average VASP PRI-values of 74% before PCI, 74% after PCI and 56% the day after PCI, as shown in Table 2. The average VASP-PRI value the day after PCI was significantly lower than VAS-PRI values pre- and post-PCI (p < 0.001). No statistically significant difference was noted between VASP PRI-values pre-PCI and post-PCI (Figure 2). A total of 32% in the *clopidogrel upstream* group reached a VASP-PRI value of less than 50% the day after PCI. In the *clopidogrel upstream* group time-separation curves showed a weak linear association between time and clopidogrel response (Figure 3) with a model coefficient of determination (r^2^) of 0.17. An average time of 16.7 hours was noted between clopidogrel administration until a VASP-PRI value of 50% was reached according to the equation outlined in Figure 3. The rate of major in-hospital bleeding was 4%.

**Table 2:** **VASP-PRI data in the 5 treatment cohorts**

	Upstream clopidogrel	Upstream clopidogrel-prasugrel switch	Prasugrel cath lab	Upstream clopidogrel-ticagrelor switch	Upstream ticagrelor
Pre-PCI VASP	74% (SD 19)	79% (SD 13)	80% (SD 15)	79% (SD 16)	64% (SD 29)
Post-PCI VASP	74% (SD 20)	74% (SD 21)	69% (SD 34)	77% (SD 20)	53% (SD 30)
Day after PCI VASP	56% (SD 27)	17% (SD 21)	19% (SD 18)	15% (SD 8)	29% (SD 25)
Percentage of patients with VASP-PRI <50% day after PCI	32%	90%	91%	100%	83%

VASP; vasodilator-stimulated phosphoprotein, PCI; Percutaneous Coronary Intervention.

### *Prasugrel*treated patient groups

**Figure 4:**
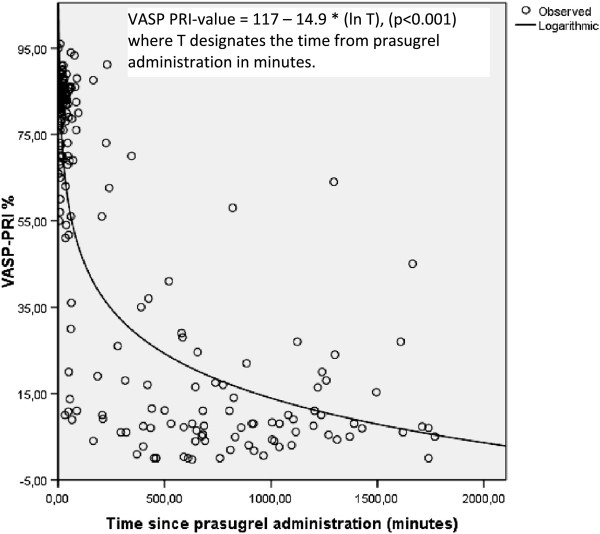
**Regression curve upstream clopidogrel-prasugrel cath lab.** VASP values as function of time in upstream clopidogrel-prasugrel cath lab patients with a logarithmic regression plot.

The average VASP-PRI values for the *upstream clopidogrel*-*prasugrel switch* cohort were 79% before PCI, 74% after PCI and 17% the day after PCI, as shown in Table 2. A statistically significant reduction was noted between VASP-PRI values pre- and post-PCI (p = 0.01) as well as between VASP PRI-values the day after PCI compared to pre- and post-PCI (p < 0.001, Figure 2). A total of 90% of patients reached the pre-specified cutoff value of VASP-PRI <50% the day after PCI (“good responders”). In time-separation curves for the *upstream clopidogrel-prasugrel switch* group (Figure 4), VASP-PRI appeared to follow an inversely logarithmic association with time, with an r^2^ of 0.66. Derived from the equation outlined in Figure 4 the average time from prasugrel administration to a VASP PRI-value of 50% was 90 minutes. The rate of major in-hospital bleeding was 1.1% in this cohort.

The average VASP-PRI values for the *prasugrel cath lab* cohort were 80%, 69% and 19%, as shown in Table 2. A significant reduction in VASP-PRI values the day after PCI compared to pre- and post-PCI values was noted (p < 0.01, Figure 2). A trend towards a reduction in VASP-PRI values pre- and post-PCI was noted, however not achieving statistical significance (p = 0.24). A total of 91% of patients managed to reach the pre-specified cutoff value of VASP PRI <50% the day after PCI. No patients experienced any major in-hospital bleedings. Too few values were obtained in the *prasugrel cath lab* group to allow a curve fit of adequate power.

### *Ticagrelor*treated patient groups

**Figure 5:**
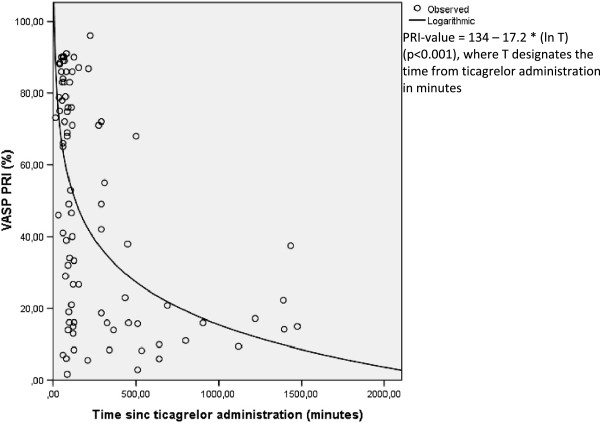
**Regression curve upstream ticagrelor.** VASP values as function of time in upstream ticagrelor patients with a logarithmic regression plot.

The average VASP-PRI values for the *upstream ticagrelor* cohort were 64% before PCI, 53% after PCI and 29% the day after PCI, as shown in Table 2. A statistically significant reduction was noted between VASP-PRI values pre- and post-PCI (p = 0.01) as well as between VASP PRI-values the day after PCI compared to pre- and post-PCI (p < 0.001, Figure 2). A total of 83% of patients reached the pre-specified cutoff value of VASP-PRI <50% the day after PCI. In time-separation curves for the *upstream ticagrelor* group (Figure 5), VASP-PRI appeared, just as in *upstream clopidogrel-prasugrel switch* patients to follow an inversely logarithmic association with time, with an r^2^ of 0.32. Derived from the equation outlined in Figure 5 the average time from ticagrelor administration to a VASP PRI-value of 50% was 2.2 hours in patients receiving upstream ticagrelor. The rate of major in-hospital bleeding was 3.3% in *upstream ticagrelor* treated patients.

In *upstream clopidogrel-ticagrelor switch* patients the average VASP PRI-values were 79%, 77% and 15% (Table 2). No difference in VASP-PRI values were noted between pre- and post-PCI. A significant reduction was noted post-PCI with all patients in this group being responders the day after PCI. No major in-hospital bleedings were noted for these patients. Too few values were obtained in the *upstream clopidogrel-ticagrelor switch* group to allow a curve fit of adequate power.

### Comparisons between treatment groups

For VASP PRI-values pre-PCI, patients treated with upstream ticagrelor had numerically lower values compared to all other groups, but the difference was not statistically significant. Post-PCI the *upstream ticagrelor* group had statistically significantly lower VASP-PRI values compared to all other groups and 50% of upstream ticagrelor patients had achieved a VASP-PRI value of <50% post-PCI. However the day after PCI, prasugrel patient groups showed lower VASP-PRI values compared to *upstream ticagrelor* as shown in Table 2. Between 83-100% of patients in the prasugrel or ticagrelor groups were responders the day after PCI, in comparison to 32% in the pre-hospital clopidogrel group (p < 0.001).

## Discussion

The main findings of our study were:

1. In STEMI patients undergoing primary PCI and given upstream clopidogrel, a switch to prasugrel in the cath lab (after coronary angiography) led to similar rates of platelet inhibition the day after PCI as prasugrel mono-therapy given in the cath lab.
2. STEMI patients given ticagrelor or prasugrel had a high degree of platelet inhibition the day after PCI with rates between 80-100% of patients being good responders according to the high on treatment reactivity definition of VASP <50%. However patients given prasugrel showed a higher degree of platelet inhibition than patients given ticagrelor mono-therapy.
3. Patients given upstream treatment with a modern P2Y12-inhibitor (in our study only ticagrelor was used upstream) had in 50% of cases adequate platelet inhibition at the time of PCI completion.
4. Time taken from prasugrel administration (on top of upstream clopidogrel) to an average VASP PRI-value of <50% was 1.5 hours. Corresponding number for ticagrelor was 2.2 hours. Clopidogrel mono-therapy was associated with a slow and heterogeneous onset of action with an average of 16.7 hours from drug administration to a VASP PRI-value of <50%.

### *Upstream clopidogrel*patients

Our study showed in the *upstream clopidogrel* group a markedly slow anti-platelet response, a finding shown in previous STEMI trials with clopidogrel [19]. A majority of patients did not achieve a VASP-PRI value of <50% the day after PCI (Figure 2 and Table 2) and the mean time to reach 50% VASP-PRI was 16.7 hours. These results differ from more stable patient populations or in non-ST-elevation acute coronary syndromes where a 600 mg loading dose of clopidogrel was associated with a more rapid, albeit slower than prasugrel, anti-platelet response [20–22]. This probably reflects (as mentioned previously) the physical stress that STEMI patients are exposed to [23]. *Clopidogrel upstream* patients showed in addition to a general slow-onset of action also significant heterogeneity in response with an overall weak linear response. Subsets of patients showed remarkable early effect, whereas other patients showed marked little response over time (Figure 3). These results are in accordance with our current state of knowledge of clopidogrel response, where patients due to several factors, both genetic as well acquired factors like diabetes, exhibit a high degree of variability in clopidogrel response, associated with different clinical outcomes [24].

### *Prasugrel*treated patients

Current guidelines suggest a loading dose of a P2Y12-inhibitor as soon as possible in the setting of STEMI undergoing primary PCI [5, 25]. In the TRITON trial, the vast majority of patients were included after a coronary angiogram was performed and no large published data exist regarding the clinical effects of upstream prasugrel treatment in STEMI patients [1]. Our study did not include prasugrel upstream since at the time of prasugrel introduction in Sweden this was not endorsed by either international or national guidelines. However our data indicated approximately 1.5 hours from prasugrel administration to a platelet “good responder” status in time separation curves. In previous studies in stable patients receiving prasugrel, approximate mean time to a 50% VASP-PRI value was only 30 min [22]. This constitutes a considerably faster onset than in our study and is probably explained by that STEMI patients are under formidable body stress, receive platelet inhibitors in supine position and are treated with opiates, all of which reduce gastrointestinal motility and uptake [19, 23].

Our data further suggested that a treatment regimen of upstream clopidogrel followed by use of prasugrel after performed coronary angiogram led to a similar degree of platelet inhibition as if giving only prasugrel in the cath lab. No tendencies for too powerful anti-platelet effects or for attenuation of anti-platelet effect (as has been suggested for cangrelor) were noted compared to prasugrel mono-therapy [26]. Major in-hospital bleeding rate for this switch group was low (1.1%). These data suggest that for patients who are given early clopidogrel, an additional bolus dose of prasugrel after coronary angiography is a pharmacodynamically feasible option making it possible to pre-treat all patients upstream with a low risk and then individualize treatment in cath lab dependening on risk-benefit ratio. This is of interest since in several countries neither prasugrel nor ticagrelor are available pre-hospitally but clopidogrel in general is. Furthermore addition of prasugrel in the cath lab led to improved platelet inhibition during PCI, although in the majority of patients not reaching a VASP-PRI value of <50% during PCI.

In a subgroup analysis of the TRITON trial, a significant and marked reduction in VASP PRI-value was noted for prasugrel 1–2 hours post loading dose compared to clopidogrel, results similar to ours. However the patients were not exclusively STEMI patients and dual treatment with clopidogrel and prasugrel was not reported [27]. In the FABOLUS PRO trial, prasugrel alone (n = 52 for the prasugrel-only group) did not achieve sufficient levels of platelet inhibition during the first 2 hours in STEMI patients undergoing primary PCI. Since no further measurements were made until 6 hours post loading, the exact time point where prasugrel alone rendered sufficient degree of platelet inhibition was not known. Our results indicate a general faster onset of prasugrel and the differences could possibly be explained by usage of different techniques for measurement of platelet aggregation (light transmission aggregometry versus VASP) with different cut-off values for adequate degree of platelet inhibition [13]. Furthermore the majority of prasugrel patients in our study were pre-treated with clopidogrel compared to the FABOLUS PRO trial [13]. In a recent study (n = 27), data indicated that the majority of prasugrel patients were responders after 2 hours (between 65%-80% of patients depending on the method of measurement). These data are in accordance with our results [12]. However a second recent study (n = 25) showed lower levels of responder rate at 2 hours (approximately 45%) [28].

### *Ticagrelor*treated patients

Our study showed that the time from upstream ticagrelor administration to an average VASP PRI-value of <50% was 2.2 hours. Like prasugrel, this constitutes a significantly slower response compared to stable patients [29]. A previous study in STEMI patients (n = 28 for ticagrelor) demonstrated that a majority of ticagrelor patients were responders after 2 hours (54%-68% depending on the method of measurement), results close to ours [12]. However ticagrelor was only given in the catheterization laboratory in that study. In another recent study (n = 25), 40% of STEMI patients given ticagrelor (in the emergency room or in the cath lab) were responders after 2 hours, results close to ours [28]. Our study showed that if ticagrelor was given very early upstream (most patients given ticagrelor in the ambulance or referring hospital) 50% were good responders at the completion of PCI (which corresponded to an average time of 2.2 hours after drug intake).

Switching from upstream clopidogrel to ticagrelor in the cath lab led to a lesser degree of platelet inhibition but well within the margin for “good responder status” compared to ticagrelor monotherapy. These data suggest, like in prasugrel treated patients, that switching from a low risk upstream option of clopidogrel followed by ticagrelor in cath lab depending on the results of the coronary angiography and after patient assessment is pharmacodynamically feasible. No major in-hospital bleeding was noted in the switch group.

### Limitations

As registry study of three drugs with different contraindications and combinations, direct clinical comparisons were not performed due to the inherent risk of selection bias. Furthermore our study sample sizes for the *prasugrel cath lab* and the *upstream clopidogrel-ticagrelor switch* groups were limited in size. Interpretation of data from these groups should be done with caution. Bleeding was used as a safety end-point, but has to be interpreted with caution and due to non-randomized data with few events no comparisons in bleeding events between groups were made. Having a group with prasugrel given upstream would have yielded further information; however at the time of prasugrel introduction in Sweden, upstream prasugrel was not endorsed in either international or national Swedish guidelines since the vast majority of patients in the TRITON trial were given prasugrel only after coronary angiography (with high CABG bleeding rates for the prasugrel arm) [1].

## Conclusions

In STEMI patients undergoing primary PCI, a switch to 60 mg prasugrel in the cath lab on top of previous upstream clopidogrel 600 mg (n = 97), led to similar rates of platelet inhibition as prasugrel mono-therapy (n = 11) with a low in-hospital bleeding rate. Patients treated with prasugrel or ticagrelor demonstrated potent anti-platelet effects with 83-100% of patients being good responders the day after PCI compared to only 32% in patients receiving only clopidogrel. Upstream treatment with ticagrelor was associated with 50% of patients being good responders at the completion of PCI.

## Funding

This was an investigator initiated study where the costs for platelet measurements were partly financed by an unrestricted grant from Eli Lilly.
